# Uncovering subclinical cardiotoxicity across chemotherapy phases in pediatric oncology

**DOI:** 10.3389/fonc.2025.1623081

**Published:** 2025-09-25

**Authors:** Esther Aurensanz-Clemente, Patricia Garcia-Canadilla, Maite Gorostegui, Cristina Rivera, María Clara Escobar-Díaz, Paula Randanne, Andres Morales La Madrid, Joan Sanchez-de-Toledo

**Affiliations:** ^1^ Department of Pediatric Cardiology, Hospital Sant Joan de Déu, Esplugues de Llobregat, Spain; ^2^ Cardiovascular Research Group iCare4Kids, Institut de Recerca Sant Joan de Déu, Esplugues de Llobregat, Spain; ^3^ Data and Digital Strategy Department, Hospital Sant Joan de Déu, Esplugues de Llobregat, Spain; ^4^ Department of Pediatric Oncology, Barcelona Pediatric Cancer Center, Hospital Sant Joan de Déu, Esplugues de Llobregat, Spain; ^5^ Department of Neonatology, Barcelona Pediatric Cancer Center, Hospital Sant Joan de Déu, Esplugues de Llobregat, Spain; ^6^ Departament of Critical Care Medicine, University of Pittsburgh, Pittsburgh, PA, United States

**Keywords:** cardiotoxicity, pediatric cancer, chemotherapy, ventricular dysfunction, echocardiography

## Abstract

**Background and objectives:**

Advances in cancer therapies have significantly improved survival rates in children. However, treatment-related toxicities remain common. This study aims to evaluate the incidence and characteristics of cardiotoxicity in a pediatric cancer cohort.

**Methods:**

This prospective study included pediatric patients who received chemotherapy between September 2020 and March 2023. Patients were categorized into five groups according to treatment phase: baseline, early treatment, late treatment, end-of-treatment, and relapse. Cardiovascular evaluation included anthropometric assessment, laboratory biomarkers, electrocardiogram (ECG) and functional echocardiography. Patients were stratified for cardiotoxicity according to pediatric and adult clinical practice guidelines.

**Results:**

265 patients were included (mean age 9.95 ± 5.26 years). The incidence of ventricular dysfunction was 2.3%. A decline in LVEF > 10% from baseline was observed in 16.5% of patients. Abnormal global longitudinal strain (GLS) values were found in 34.7%; significant ECG changes in 16.2%, and elevated Troponin I levels in 7.1%. Based on echocardiographic and laboratory findings, patients undergoing treatment showed greater cardiac involvement compared with those in other groups.

**Conclusions:**

Although the overall incidence of overt ventricular dysfunction was low, the use of ECG and GLS enhanced the sensitivity for detecting of subclinical cardiac impairment in pediatric patients receiving chemotherapy.

## Introduction

Cancer is the leading cause of disease-related mortality in the pediatric age group (1). Thanks to advances in the diagnosis and treatment of childhood cancer, more patients are now surviving to adulthood. However, this improved survival has also brought to light the substantial burden of cardiovascular disease, with increased morbidity and mortality attributed to cardiotoxicity (2, 3).

Childhood cancer survivors (CCS) face a significantly elevated risk of cardiovascular disease as a late effect of cancer therapy. According to data from the Childhood Cancer Survivor Study (CCSS), the risk of developing cardiovascular disease is 5 to 15 times higher in CCS compared to the general population, depending on the specific malignancy and treatment exposure. Moreover, the risk of heart failure is up to 8 times greater in CCS than in their healthy siblings. The spectrum of cardiotoxicity-related complications is broad and includes cancer therapy-related cardiac dysfunction (CTRCD), arrhythmias, valvular disease, and pericardial involvement. Among these, CTRCD is one of the most common and clinically significant. Despite its potential reversibility with early detection and intervention, it often remains undiagnosed in its subclinical stages (4–10).

Cardiotoxicity can manifest in the short, medium, or long term (11). Its etiology and pathogenesis are multifactorial, influenced by both the underlying disease and the treatment received. Moreover, patient-specific risk factors, including individual predisposition and lifestyles, also modulate the development of cardiotoxicity (12).

Currently, the evaluation of cardiovascular function is mostly based on echocardiography and the analysis of serum biomarkers such as Troponin or the N-terminal pro b-type natriuretic peptide (NT-proBNP) (10). Myocardial function is easily assessed using echocardiography. Particularly useful for the quantification of the myocardial function are the left ventricular ejection fraction (LVEF) and the Global Longitudinal Strain (GLS) (13, 14) an echocardiographic measure of myocardial deformation that is more sensitive than LVEF in the detection of subclinical dysfunction or asymptomatic CTRCD (10, 13–15)^3^. Current guidelines establish a cardiotoxicity risk stratification by measuring of LVEF and GLS, and assessing their decline from baseline.

Follow-up recommendations for pediatric cancer survivors have been recently published by the American Society of Echocardiography (16). According to these guidelines, an LVEF greater than 55% is considered within normal limits, whereas an LVEF below 50% and/or a GLS above –16% (less negative) is classified as abnormal.

This study aimed to perform a comprehensive cardiovascular evaluation in children with cancer at different stages of their disease from diagnosis to the end of treatment.

## Materials and methods

### Study design and population

A descriptive, single-center, cross-sectional study was conducted at a tertiary, referral, pediatric cancer center in Spain. The study enrolled all consecutive children (< 18 years old) with a diagnosis of cancer who underwent cardiovascular assessment at the cardio-oncology unit between September 2020 and March 2023. Only children with a diagnosis of onco-hematological disease requiring chemotherapy were included in the study.

**Figure 1:**
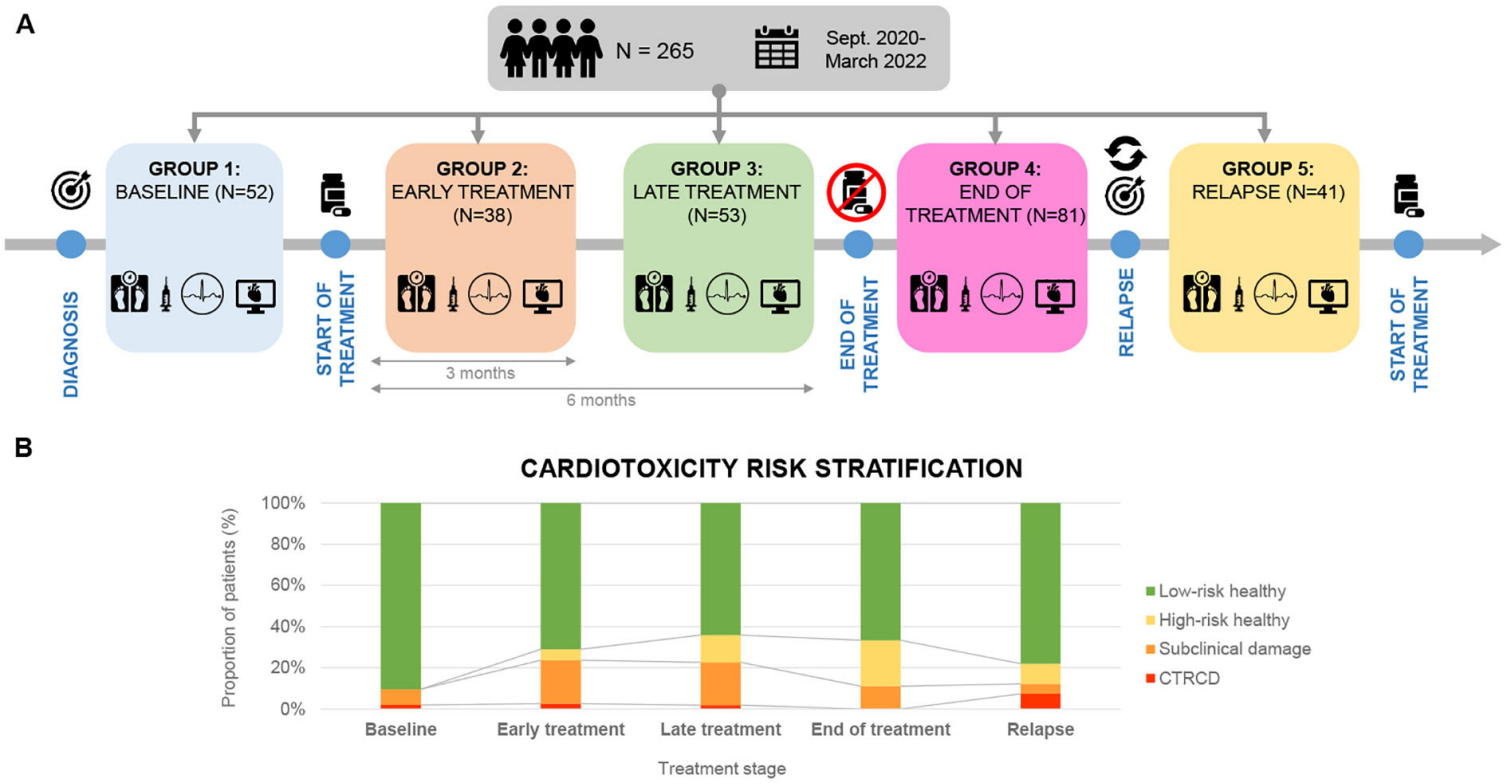
**(A)** Cross-sectional study with a sample of 265 patients recruited from September 2020 to March 2022. Patients were divided into 5 groups according to chemotherapeutic treatment phase. **(B)** Cardiotoxicity risk classification in each treatment phase (Ventricular dysfunction due to cardiotoxicity- Subclinical damage- High risk healthy- Low risk healthy).

Patients were included at different disease stages and assessed at a single point in time without follow-up. Thus, five different cohorts were defined according to their stage of treatment (**Figure 1**): i) *Baseline*: before the initiation of chemotherapy*; ii) Early treatment*: 3 months after initiation of treatment; iii) *Late treatment*: 6 months after the initiation of treatment; iv) *End-of-treatment*: 2 months after the end of treatment; *v) Relapse:* assessment at the time of relapse before the initiation of new onco-hematological treatment.

### Study variables

A complete medical history and physical examination were performed, including the determination of body mass index (BMI) and blood pressure (BP) with respective Z score values. Overweight was defined as BMI ≥ 2 standard deviations (SD) and underweight as BMI < 2 SD. Systolic hypertension was defined as systolic blood pressure (SBP) ≥ 2 SD and diastolic hypertension as diastolic blood pressure (DBP) ≥2 SD. Data regarding chemo- and radiotherapy were collected. Patients receiving anthracycline doses greater than >249 mg/m2 and/or chest radiotherapy over15Gy were further classified as High-Risk.

**Figure 2:**
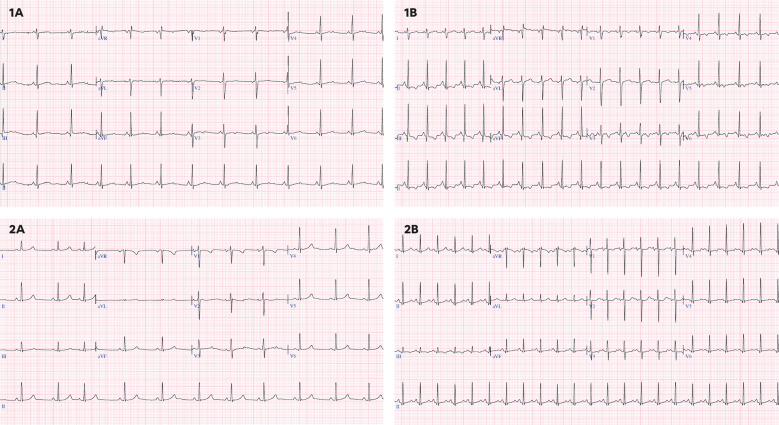
**(1A)** 16 years old, baseline ECG with sinus rhythm at 73 bpm, QTc(B) 441 ms, QTc(F) 427 ms without repolarization disturbance. **(1B)** 16 years old, ECG under chemotherapy with sinus rhythm at 118 bpm, QTc(B) 448 ms, QTc(F) 400 ms with repolarization alteration and negative T waves in II, III, aVF, V5,V6. **(2A)** 12 years, baseline ECG with sinus rhythm at 66 bpm, QTc(B) 377 ms, QTc(F) 371 ms, without repolarization alteration. **(2B)** 12 years, ECG under chemotherapy with sinus rhythm at 136 bpm, with QT segment lengthening and asymmetric T-wave rise with QTc(B) 481 ms, QTc(F) 420 ms, without repolarization changes.

In addition, the following complementary tests were performed: 12-lead electrocardiogram (ECG), a functional echocardiography and, serum biomarkers. ECG included the measurement of the following parameters: heart rate (HR), PR interval (ms), QRS complex (ms), and QT interval corrected according to the Fridericia and Bazett (B) formula. Long QT was defined as a QTc (B) ≥ 450 ms. Repolarization disturbance was defined as flattening or inversion of the T wave in the left anterior precordial leads (II, III, aVF, V5, V6) (**Figure 2**).

All patients underwent a comprehensive echocardiographic study including both morphological and, functional assessment. Images were acquired and further analyzed following the institutional echocardiographic study protocol. Speckle Tracking Echocardiography (STE) method was used to measure Global Longitudinal Strain (GLS) in all study patients (**Supplementary Material 1**). In addition, all patients had a baseline echocardiography performed prior to the initiation of treatment, with LVEF measured at that time. This baseline value was used to determine whether a >10% drop in LVEF had occurred. Troponin I and NT-proBNP levels were measured in all study patients. Both biomarkers were analyzed using a chemiluminescent microparticle immunoassay (CMIA) on the Alinity analyzer platform (Abbott Laboratories, Abbott Park, IL, USA). Using the aforementioned Cardioongology guidelines, with the caveat of not having baseline measurements of the cardiac function, four different groups of cardiovascular function were defined for this study (16). (**Figure 1**): Group A (*CTRCD*): LVEF <55%, Group B (*subclinical damage)*: LVEF >55% and/or positive Troponin I, Group C (*high-risk healthy)*: LVEF >55% and negative Troponin I, Group D (*low-risk* healthy*)*: LVEF >55% and negative Troponin I.

### Statistical analysis

Statistical analysis of the data was performed with SPSS 28.0 for Windows. Normality was tested according to the Kolmogorov-Smirnov and Shapiro-Wilk criteria. Differences between groups were analyzed for statistical significance using one-way ANOVA followed by *post-hoc* Tukey test for all pairwise comparisons. To perform hypothesis testing on qualitative categorical variables, the χ2 test was used. In all analyses, a p-value <0.05 was considered statistically significant.

### Ethical aspects

Informed consent was obtained from patients/legal guardians before their inclusion in the study. The project developed according to the Declaration of Helsinki in its latest revision of 2013 and under the guidelines of the Law 14/2007 on Biomedical Research. Furthermore, the study was approved by the Sant Joan de Déu Foundation Research Ethics Committee and the processing, communication, and transferring of personal data of all participants complies with current legislation (European Regulation EU2016/679 and Organic Law3/2018 of 5 December on the Protection of Personal Data): PIC-227-19; PIC-88-24.

## Results

### Clinical and laboratory variables

Two hundred sixty-five patients were analyzed, of whom 156 (58.8%) were male. Their mean age at enrollment was 9.95 ± 5.26 years. There were no significant differences in BMI across groups, with 4.5% (12/265) of patients classified as overweight and 4.9% (13/265) as underweight. The overall incidence of systolic hypertension was 17/265 (6.41%).

The most common diagnoses included: leukemia: 110 (41.5%), lymphoma: 53(20%), bone sarcomas: 39 (15%), kidney tumors 15 (5.5%) and CNS tumors: 13 (5%) (**Table 1**). Only 5/265 (1.9%) patients received cardiovascular medications such as ACE inhibitors or beta-blockers, and 4/265 (1.5%) dexrazoxane for cardioprotection. Patients were allocated to the five different groups as follows: baseline, 52/265 (19.6%); early treatment, 38/265 (14.3%); late treatment, 53/265 (20%); end-of-treatment, 81/265 (30.6%); relapse, 41/265 (15.5%).

**Table 1:** The cohort’s tumor distribution.

	Total=265
Leukemias	**110 (41.5%)**
I ALL	1
B ALL	85
T ALL	11
AML	13
Lymphoma	**53 (20%)**
Burkitt lymphoma	11
Hodgkin lymphoma	38
Non-Hodgkin lymphoma	4
Bone sarcoma	**39 (15%)**
Ewing sarcoma	24
Osteosarcoma	15
Kidney tumor	**15(5.5%)**
Kidney tumor	3
Wilms tumor	12
CNS Tumor	**13(5%)**
Astrocitoma	2
ATRT	2
Glioma	4
Meduloblastoma	4
Craniofaringioma	1
Soft tissue sarcoma	**7(2.5%)**
Soft tissue sarcoma	7
Germ-cell tumor	**6(2.3%)**
Germ-cell tumor	6
Hepatoblastoma	**4(1.5%)**
Hepatoblastoma	4
Other	**18(6.8%)**
Neuroblastoma	10
Carcinoma	2
MPNST	2
Coriocarcinoma	1
Lung Blastoma	1
Peritoneal mesothelioma	1
Retinoblastoma	1

ALL, Acute lymphoblastic Leukemia; B, B-lymphocyte; T, T-lymphocyte; AML, Acute myeloid leukemia; ATRT, Atypical teratoid rhabdoid tumor; MPNST, Malignant Peripheral Nerve Sheath Tumors.

The bold highlights the main diagnoses.

The cumulative anthracyclines doses and radiotherapy exposure at the end-of-treatment of each of the study groups is detailed in **Table 2**.

**Table 2:** Analysis of anthropometric variables in the different groups according to the treatment phase.

	Baseline n=52	Early treatment n=38	Late treatment n=53	End of treatment n=81	Relapse n=41	P
Age (years)	8.08 ± 5.45	8.68 ± 5.24	8.83 ± 5.08	12.64 ± 4.46	9.62 ± 4.88	**0.001**
Sex (M/F)	31/21 (59.6/40.4%)	15/23 (39.5/60.5%)	35/18 (66/34%)	48/33 (59.3/40.7%)	27/14 (65.9/34.1%)	0.096
Weight (Z)	0.31 ± 1.21	-0.08 ± 1.15	-0.05 ± 1.13	0.19 ± 1.04	-0.29 ± 1.21	0.086
Height (Z)	0.6 ± 1,15	0.01 ± 1.13	0.32 ± 1.32	-0.18 ± 1.11	-0.46 ± 1.23	**0.007**
BMI (Z)	0.23 ± 1.28	-0.07 ± 1.29	-0.19 ± 1.33	0.30 ± 1.04	0.02 ± 1.15	0.132
Overweight (BMI Z≥2SD)	5/52 (9.6%)	2/38 (5.3%)	2/53 (3.8%)	3/81 (3.7%)	0/41 (0%)	0.050
Underweight (BMI Z <-2SD)	1/52 (1.9%)	1/38 (2.6%)	6/53 (11.3%)	1/81 (1.2%)	4/41 (9.8%)	0.050
SBP (Z)	0.76 ± 1.15	0.47 ± 1.16	0.26 ± 1.04	-0.02 ± 0.91	-0.17 ± 1.09	**0.001**
DBP (Z)	1.55 ± 0.95	1.63 ± 0.95	1.38± 0.99	1.10 ± 0.95	1.17 ± 1.00	**0.020**
SBP (Z≥2SD)	6/51 (11.8%)	4/38 (10.5%)	3/51 (5.9%)	1/77 (1.3%)	3/40 (7.5%)	0.150
DBP (Z≥2SD)	17/51 (34%)	13/38 (34.2%)	17/51 (33.3%)	11/77 (14.3%)	12/40 (30%)	**0.049**
Troponin (ng/L)	0.003 ± 0.005	0.012 ± 0.014	0.006 ± 0.008	0.003 ± 0.005	0.002 ± 0.005	**<0.001**
Troponin (>0.020 ng/ml)	1/47 (2.1%)	5/24 (20.8%)	5/44 (11.4%)	3/68 (4.4%)	1/27 (3.7%)	**0.027**
proBNP	337.31 ± 591.85	104.13 ± 73.01	226.02 ± 748.17	70 ± 51.80	166.76 ± 226.23	**0.030**
Urea(mg/dL)	20.17 ± 0.99	19.92 ± 0.14	26.20 ± 0.25	28.05 ± 0.12	22.48 ± 0.12	**0.004**
Creatinine (mg/dL)	0.45 ± 0.022	0.47 ± 0.016	0.49 ± 0.026	0.61 ± 0.023	0.51 ± 0.023	0.084
AST (UI/L)	53.69 ± 75.56	45.08 ± 78.82	32.94 ± 22.19	27.39 ± 14.28	37.28 ± 24.32	0.070
ALT (UI/L)	72.38 ± 135.69	75.89 ± 143.61	55.35 ± 70.11	40.72 ± 71.38	39.20 ± 32.11	0.254
Anthracycline (>249 mg/m2) *	28/52 (53.8%)	21/38 (55.3%)	31/53 (58.5%)	25/81 (30.9%)	18/41 (43.9%)	**0.009**
Chest Radiotherapy (>15 Gy)*	9/52 (17.3%)	11/38 (28.9%)	16/53 (30.2%)	21/81 (25.9%)	17/41 (41.5%)	0.153

BMI, Body mass index; SBP, Systolic blood pressure; DBP, Diastolic blood pressure; AST, aspartate aminotransferase; ALT, Alanine aminotransferase; SD, standard deviation; Z, Z-score, p value <0.05 statistically significant, *The cumulative anthracyclines doses and radiotherapy exposure at the end-of-treatment of each of the study groups.

The bold highlights the statistical significance.

### Serum biomarkers

Troponin I: Elevated levels were observed in 7.1% of patients, with the highest values in the early treatment group (0.012 ± 0.014 ng/ml; p < 0.001). The percentage of patients with high troponin levels was significantly higher in the early and late groups compared to the end-of-treatment group (20.8 *vs* 11.4 *vs* 4.4%; p=0.027).

NT-proBNP: The highest values were found in the baseline group, while the lowest levels seen in those of the end-of-treatment group (337.31 ± 591.85 *vs* 70 ± 51.80 ng/L; p=0.030).

### Electrocardiographic variables

Electrocardiographic abnormalities were identified in 16.2% of patients. Prolonged QTc (QTc > 450 ms) was observed in 12.4% (33/265) of patients (**Table 3**). Compared to the baseline group, a significantly higher proportion of patients in the early and late treatment groups had prolonged QTc (0% *vs*. 21.1% *vs*. 15.1%; p = 0.007). Patients in the early and late groups had significantly longer QTc intervals than those in the baseline group, regardless of whether Bazett’s (417.08 ± 36.51; 420.08 ± 27.45 *vs*. 392.78 ± 29.62 ms; p = 0.001) or Fridericia’s correction was applied (389.05 ± 29.93; 393.86 ± 24.59 *vs*. 377.51 ± 27.22 ms; p = 0.034). Repolarization abnormalities were present in 8.7% (23/265) of patients, defined by flat or negative T waves in left precordial leads. Compared to baseline, both early and late treatment groups had a significantly higher proportion of patients with repolarization abnormalities (3.8% *vs*. 13.2% *vs*. 18.9%; p = 0.007). Although statistically significant differences were found in PR interval and QRS duration between groups, all values remained within normal limits.33/265 (12.4%).

**Table 3:** Analysis of electrocardiographic variables in the different study groups.

n= 265	Baseline n= 52	Early treatment n= 38	Late treatment n= 53	End of treatment n= 81	Relapse n= 41	P
HR (bpm)	93.04 ± 31.59	99.60 ± 26.73	92.08 ± 20.06	75.32 ± 16.61	88.68 ± 17.72	<0.001
PR (ms)	124.08 ± 20.10	117.94 ± 17.37	125.36 ± 16.44	131.28 ± 17.50	126.79 ± 18.66	0.006
QRS (ms)	82.37 ± 16.32	80.91 ± 11.21	82.64 ± 11.76	88.49 ± 10.79	82.84 ± 13.95	0.009
QTc^B^ (ms)	392.78± 29.62	417.08 ± 36.51	420.08 ± 27.45	404.89 ± 33.99	407.50 ± 40.73	<0.001
QTc^F^(ms)	377.51± 27.22	389.05 ± 29.93	393.86 ± 24.59	380.61 ± 33.25	378.36 ± 39.21	0.034
QTc^B^ >450ms	0/52 (0%)	8/38(21%)	8/53 (15%)	12/81 (15%)	5/41 (12%)	0.007
Rep abn	2/52 (4%)	5/38 (13%)	10/53 (19%)	2/81 (2.5%)	4/41 (10%)	0.007

HR, Heart rate; QTc^B^, QTc Bazett; QTc^F^, QTc Fridericia; Rep abn, repolarization abnormalities, *p* value <0.05 statistically significant.

### Echocardiographic variables

#### Left ventricular systolic function

The overall incidence of CTRCD was 2.3%. Patients in late treatment group had a lower LVEF compared to the baseline group (66.30 ± 5.60 *vs* 69.43 ± 5.74%; p=0.025); 16.5% presented a >10% drop in LVEF from baseline, but no significant differences were found between groups (**Table 4**). In 34.7% of cases, GLS was ≤18%. Although the early and late groups had a higher proportion of patients with reduced GLS than the baseline group, these differences were not statistically significant. LV dilatation was observed in 6.8%, with the largest dimensions found in the baseline and late groups compared to the end-of-treatment group (Z-score: 0.39 ± 1.55 *vs*. 0.00 ± 1.37 *vs*. -0.87 ± 1.42; p = 0.022).

**Table 4:** Analysis of echocardiographic variables in the different study groups.

n= 265	Baseline	Early treatmet	Late treatmet	End of treatmet	Relapse	P
	n= 52	n= 38	n= 53	n= 81	n= 41	
Left ventricle: systolic function						
LVEDD (Z)	-0.39 ± 1.55	-0.29 ± 1.71	0.00 ± 1.37	-0.87 ± 1.42	-0.46 ± 1.52	0.022
IVSd (Z)	-0.41 ± 1.34	-0.54 ± 1.85	-0.45 ± 1.83	-0.27 ± 1.08	-0.19 ± 1.12	0.781
LVPWd (Z)	-0.44 ± 1.09	-0.49 ± 1.46	-0.89 ± 1.48	-0.85 ± 1.33	-0.45 ± 1.22	0.181
SF (%)	41.65 ± 5.99	38.56 ± 4.37	38.16 ± 4.28	39.38 ± 5.43	38.10 ± 5.98	0.005
EF (%)	72.79 ± 6.90	69.35 ± 5.17	68.57 ± 5.53	69.99 ± 6.66	68.25 ± 7.85	0.005
LVEF S (%)	69.43 ± 5.74	69.54 ± 5.39	66.30 ± 5.60	67.22 ± 5.78	67.53 ± 7.21	0.025
LVEF S<55%	1/51 (1.9%)	1/38 (2.7%)	1/53 (1.9%)	0/81 (0%)	3/41 (7.3%)	0.155
ΔLVEF (%)	–	-0.70 ± 10.99	-3.64 ± 8.52	-3.44 ± 15.56	-4.82 ± 9.32	0.593
ΔLVEF >10%	–	6/33 (18.2%)	9/43 (20.9%)	21/71 (29.6%)	8/28 (28.6%)	0.582
CO (ml/m2)	2.85 ± 1.37	2.91 ± 1.48	2.98 ± 1.40	2.59 ± 1.12	2.63 ± 1.50	0.456
MAPSE (mm)	13.72 ± 3.47	12.73 ± 3.12	13.5 ± 3.48	14.36 ± 3.55	12.36 ± 2.97	0.026
SAPSE (mm)	13.24 ± 3.89	12.24 ± 2.87	12.94 ± 2.97	14.47 ± 3.18	13.20 ± 3.49	0.010
S’lat TDI	7.73 ± 1.86	8.25 ± 2.08	7.71 ± 1.94	8.61 ± 2.12	7.84 ± 3.03	0.096
S’med	6.94 ± 1.64	7.12 ± 1.41	6.64 ± 1.15	6.87 ± 1.22	6.53 ± 1.51	0.311
GLS (%)	-21.81 ± 2.97	-20.06 ± 9.73	-19.31 ± 7.34	-20.97 ± 2.61	-20.39 ± 0.55	0.379
GLS ≤ 18%	9/49 (18.4%)	9/34 (26.5%)	14/51 (27.5%)	18/78 (23.1%)	9/37 (24.3%)	0.855
Left ventricle: diastolic function						
LA (ml/m2)	13.23 ± 6.14	11.33 ± 5.98	13 ± 5.17	11.65 ± 4.38	11.14 ± 6.24	0.182
LA >16 ml/m2	15/52 (28.9%)	8/38 (21%)	10/53 (18.9%)	11/81 (13.6%)	6/41 (14.6%)	0.243
E/A ratio MV	2.15 ± 0.96	1.47 ± 0.37	1.68 ± 0.38	2.08 ± 0.59	1.89 ± 0.56	<0.001
E/E’ lat	6.67 ± 2.40	6.21 ± 1.98	6.68 ± 1.93	5.51 ± 2.00	5.93 ± 1.79	0.005
E’/A’ lat	2.54 ± 1.03	2.38 ± 0.83	2.41 ± 0.93	2.88 ± 1.23	2.56 ± 0.83	0.058
E/E’ med	8.36 ± 2.87	7.88 ± 1.82	9.13 ± 2.29	7.85 ± 2.25	8.34 ± 2.25	0.046
E’/A’ med	2.37 ± 0.79	1.99 ± 0.55	1.96 ± 0.69	2.49 ± 0.85	2.05 ± 0.63	<0.001
E/E’ average	7.55 ± 2.32	7.01 ± 1.76	7.91 ± 1.80	6.72 ± 2.11	7.14 ± 1.83	0.015
E’/A’ average	2.41 ± 0.83	2.17 ± 0.59	2.15 ± 0.73	2.64 ± 0.84	2.29 ± 0.60	0.002
Pulm veins S/D	1.07 ± 0.26	0.89 ± 0.21	0.98 ± 0.29	0.98 ± 0.31	0.97 ± 0.21	0.177
Right ventricle: systolic & diastolic function						
TAPSE (mm)	20.33 ± 4.14	18.22 ± 3.15	20.52 ± 4.62	20.93 ± 3.94	18.90 ± 4.15	0.005
E/A TV	1.71 ± 0.55	1.41 ± 0.46	1.40 ± 0.39	1.84 ± 1.89	1.33 ± 0.35	0.064
S’ TV	11.39 ± 2.74	11.05 ± 2.17	11.79 ± 2.64	11.99 ± 2.71	12.33 ± 3.77	0.302
E/E’ TV	4.51 ± 1.79	5.65 ± 1.78	4.92 ± 1.68	4.81 ± 1.93	5.31 ± 2.79	0.094
E’/A’TV	1.79 ± 0.91	1.46 ± 0.73	1.59 ± 0.61	1.80 ± 0.88	1.31 ± 0.43	0.008

LVEDD, Left ventricle end-diastolic diameter; IVSd, interventricular septum diastolic; LVPWd, left ventricular posterior wall diastolic; SF, shortening fraction; EF, Ejection fraction; LVEF S, Left Ventricular Ejection fraction measured by Simpson; ΔLVEF, Difference of LVEF from Baseline; CO, Systemic cardiac output (ml/m2); MAPSE, Mitral Annular Plane Systolic Excursion; SAPSE, Septal Annular Plane Systolic Excursion; GLS, global longitudinal strain; MV, mitral valve; TAPSE, Tricuspid Annular Plane Systolic Excursion, LA, Left atrium, TV, tricuspid valve, Pulm veins S/D. p value <0.05 is statistically significant.

#### Left ventricular diastolic function

In all groups, the E/A ratio was >1. The lowest values were seen in early group and the highest in the baseline group (1.47 ± 0.37 *vs* 2.15 ± 0.96 *vs*; p < 0.001). At the end-of-treatment, the E/E’ ratio (both lateral and medial) was significantly lower than in the late treatment group (E/E’ lateral: 5.51 ± 2.00 *vs*. 6.68 ± 1.93; p = 0.005; E/E’ medial: 7.85 ± 2.25 *vs*. 9.13 ± 2.29; p = 0.046), although all values remained within normal ranges. Left atrial dilatation was uncommon, with only one case detected with an indexed volume > 34 ml/m². The baseline group had a higher proportion of patients with left atrial dilatation than the end-of-treatment group (28.9% *vs*. 13.6%), although this was not statistically significant.

#### Right ventricular systolic function

Patients in the early group had lower TAPSE values compared to those in the end-of-treatment group (18.22 ± 3.15 *vs* 20.93 ± 3.94 mm; p=0.008).

No significant valvar insufficiencies, signs of pulmonary hypertension or pericardial effusion were observed during the echocardiographic assessments.

### Risk stratification - incidence of cardiotoxicity

There were statistically significant differences in the distribution of cardiovascular risk categories across treatment groups. A higher proportion of patients in the treatment groups had subclinical damage or high cardiovascular risk compared to the baseline group (33.3% *vs*. 5.7%; p = 0.001) (**Table 5**). When patients were classified solely by LVEF, no significant differences were observed among groups.

**Table 5:** Cardiotoxicity risk stratification (guidelines adult/pediatric).

	Baseline	Early treatment	Late treatment	End of treatment	Relapse	P
	n=52	n=38	n= 53	n= 81	n= 41	
CTRCD	1 (1.9%)	1 (2.6%)	1 (1.9%)	0	3 (7.3%)	0.001
Subclinical damage	3 (5.7%)	8 (21.1%)	11 (20.8%)	9 (11.1%)	3 (7.3%)	
High-risk healthy	0	2 (5.3%)	7 (13.2%)	18 (22.2%)	5 (12.2%)	
Low-risk healthy	48 (92.4%)	27 (71.1%)	34 (64.2%)	54 (66.7%)	30(73.2%)	

CTRCD, cancer therapy-related cardiac dysfunction. p value <0.05 statistically significant.

## Discussion

Our analysis of a large cohort of pediatric oncology patients demonstrates that cardiovascular risk stratification evolves throughout chemotherapy treatment, with a greater proportion of patients presenting subclinical myocardial damage or high cardiovascular risk during therapy. The overall incidence of CTRCD was 2.3%, but 16.5% experienced a ≥10% decrease in LVEF from their baseline, and 34.7% had abnormal GLS values, indicating subclinical dysfunction. ECG abnormalities were observed in 16.2% of patients, primarily QT prolongation and repolarization disturbances, and 7.1% had elevated troponin levels. Interestingly, patients in the early and late treatment groups showed greater cardiac involvement, as evidenced by echocardiographic and biomarker alterations. These findings reinforce the need for continuous cardiac surveillance throughout treatment to detect early cardiotoxicity and prevent irreversible damage.

The incidence of CTRCD related to cardiotoxicity in adulthood is widely reported in the scientific literature. Cardinale et al., described an incidence of cardiotoxicity of 9% in their studied sample (14). However, information in children is scarce and there is great variability depending on the population studied and the diagnostic methods used. Bu-Lock et al. (17), analyzed 125 pediatric patients and reported an incidence of CTRCD of 5% with 19.2% of patients experiencing a significant fall in LVEF. Similarly, Agha et al. (18), in a study including 40 patients, described 5% of CTRCD with 40% of the patients experiencing decrease in LVEF. However, Kocabas et al. (19), was not able to identify CTRCD in 72 patients. In our study, despite only 2.3% of patient having CTRCD, 16.5% had subclinical impairment of the myocardial function.

Traditionally, LV systolic function has been assessed using LVEF and FS, though these measures often fail to detect subtle myocardial changes, as chemotherapy-induced damage tends to be regional and asymmetric (20). More sensitive approaches, such as tissue Doppler imaging (TDI), have been suggested as potential alternatives, particularly for tracking medial S’ velocity declines during chemotherapy (17–19, 21). Although the late treatment group in our study had worse TDI values than the other groups, these differences were not statistically significant.

Systematic measurement of GLS also allows early identification of systolic function abnormalities and has been correlated with the development of long-term cardiotoxicity (22). Thavendiranathan et al., demonstrated that the early fall in GLS was more sensitive than LVEF analysis (21). In this study, a 10-15% decrease in GLS was considered the most useful parameter for predicting long-term cardiovascular disease. However, the SUCCOUR study also questions the usefulness of this parameter, finding no significant difference in patient outcomes when function assessment at follow-up was based on GLS rather than LVEF (23). In our study, GLS was altered in 34.7% of patients, significantly increasing the sensitivity for the detection of cardiovascular risk groups compared to traditional function assessment parameters. However, without longitudinal follow-up, it remains unclear how these early GLS alterations translate into long-term cardiac dysfunction in children surviving from cancer.

Diastolic dysfunction has been explored as an early marker of cardiotoxicity in several studies. To date, there is no scientific evidence to clarify the diagnostic and/or prognostic value of its assessment. Furthermore, in the pediatric age group, changes in diastolic function seem to occur later and have great variability according to age, making their use difficult (17, 24).

Right ventricular function assessment in pediatric oncology patients presents similar challenges, as classical indices such as TAPSE and RV fractional area change have limited sensitivity in detecting subtle RV dysfunction (25). In this study, no significant alterations in RV function or pulmonary hypertension were detected, suggesting that RV involvement may be less prominent in early chemotherapy exposure.

While echocardiographic parameters provide valuable insights, ECG abnormalities have also been reported in pediatric patients undergoing chemotherapy. Previous studies have described conduction disturbances, repolarization abnormalities, and QT prolongation in up to 25% of patients (18, 26). In this study, 16.2% of patients had ECG alterations, most commonly QT prolongation and repolarization disturbances, findings that were particularly pronounced in patients undergoing active chemotherapy. These results suggest that ECG monitoring could be useful for tracking transient electrophysiological changes during treatment, though the long-term significance of these findings remains to be determined.

Biomarkers such as troponin and NT-proBNP remain the most widely used markers for detecting chemotherapy-induced myocardial injury, though interest is growing in newer biomarkers such as microRNAs, and proteomics (27). Troponins, particularly troponin I and troponin T, are the gold standard for detecting myocardial necrosis. In this study, troponin elevations were found in 7.1% of patients, with the highest values observed in those currently undergoing treatment, reinforcing its role as a potential early indicator of myocardial damage (28). NT-proBNP, a well-established marker in heart failure, has also been associated with chemotherapy-induced cardiotoxicity, with levels above 100 ng/L linked to an increased risk of cardiac events (29, 30). In pediatric patients, elevated NT-proBNP levels have been correlated with CTRCD compared to healthy controls (31). Interestingly, in our study, the highest values of NT-proBNP were found in the baseline group, while the lowest levels found in those of the end-of-treatment group suggesting than other factors, such as, inflammation, fluid management, might play a role.

The International Late Effects of Childhood Cancer Guideline Harmonization Group has developed evidence-based recommendations for long-term cardiovascular monitoring in childhood cancer survivors (32). Risk stratification is primarily based on cumulative anthracycline exposure and chest radiotherapy, which determine follow-up intervals. Interestingly, when applying adult guideline criteria, a higher percentage of patients in the early (23.7%) and late (26.4%) treatment groups were classified as having subclinical myocardial dysfunction or high cardiovascular risk. These same groups also had the highest prevalence of ECG abnormalities, reinforcing the need for continuous cardiovascular monitoring throughout treatment. The findings from this study suggest that pediatric oncology patients should undergo dynamic cardiovascular risk stratification at each stage of treatment, allowing for early interventions that may help mitigate long-term cardiovascular complications.

## Conclusion

Cardiovascular function in children with cancer changes dynamically throughout chemotherapy, with significant alterations occurring during active treatment phases. Frequent cardiovascular assessments are essential for early detection of myocardial dysfunction, allowing for timely interventions to prevent irreversible cardiac damage. GLS and ECG abnormalities appear to improve sensitivity in detecting subclinical cardiotoxicity, though long-term studies are needed to confirm their prognostic value in children. Pediatric oncology patients require individualized, stage-specific cardiovascular risk stratification to optimize long-term cardiac outcomes. Future studies with larger cohorts and longitudinal follow-up are necessary to refine screening protocols and risk stratification models in pediatric cardio-oncology.

## Data Availability

The raw data supporting the conclusions of this article will be made available by the authors, without undue reservation.
