# Supplementary material for: Uncovering subclinical cardiotoxicity across chemotherapy phases in pediatric oncology
Source: Front Oncol. 2025 Sep 25;15:1623081. doi: 10.3389/fonc.2025.1623081 (PMC12507561; doi:10.3389/fonc.2025.1623081)
Supplement: Supplementary file 1 [file Table1.docx]

Supplementary file:

High-resolution functional echocardiography acquisition protocol

* Parasternal long axis and short axis views. The average of the two measurements was taken in Z score values: left ventricular end-diastolic diameter (LVEDD), interventricular septum (ISV) , and posterior wall (PW). The shortening fraction (SF) and ejection fraction (EF) were expressed as a percentage.

* left ventricular volumetric. EF was taken with the Simpson method (%) in 4 chambers. Ventricular dysfunction was considered to be Simpson LVEF < 53%. The difference

lated as baseline Simpson LVEF – Simpson LVEF at the time analyzed. A drop of > 10% in Simpson's LVEF was considered positive.

* left atrial volumetric. It was measured in plane 4 C focused on the left atrium with measurement of maximum atrial volume. It was expressed in ml/m2 and indexed by body surface area. It was classified as: LA dilated (LA > 34 ml /m2), LA slightly dilated (LA ≥ 16 ml/m2) and LA normal (LA < 16 ml /m2).

* Cardiac output (ml/m2)

* MAPSE, SAPSE, TAPSE: measured in mm. It was measured in plane 4 C with M mode.

* E wave/A wave ratio in mitral valve and tricuspid valve. It was measured at 4 C with pulsed Doppler in both valves and the average of three beats was taken.

* Tissue Doppler: lateral, medial and right ventricle were measured. Waves E', A' and S' were collected. The lateral and medial measurements of the left ventricle were averaged.

* Pulmonary vein pattern: relationship of S wave to D wave.

* Myocardial deformity study: longitudinal global strain and 4-chamber strain study were performed with TOMTEC post-processing analysis software. It was expressed in %.
